# Dissociating self-generated volition from externally-generated motivation

**DOI:** 10.1371/journal.pone.0232949

**Published:** 2020-05-19

**Authors:** Laurel S. Morris, Agnes Norbury, Derek A. Smith, Neil A. Harrison, Valerie Voon, James W. Murrough

**Affiliations:** 1 Department of Psychiatry, Depression and Anxiety Center for Discovery and Treatment, Icahn School of Medicine at Mount Sinai, New York, New York, United States of America; 2 Department of Psychiatry, Icahn School of Medicine at Mount Sinai, New York, New York, United States of America; 3 Department of Radiology, BioMedical Engineering and Imaging Institute, Icahn School of Medicine at Mount Sinai, New York, New York, United States of America; 4 Division of Psychological Medicine and Clinical Neuroscience, Cardiff University, Cardiff, United Kingdom; 5 Department of Neuroscience, Brighton and Sussex Medical School, University of Sussex, Brighton, United Kingdom; 6 Department of Psychiatry, University of Cambridge, Addenbrooke’s Hospital, Cambridge, United Kingdom; 7 Department of Neuroscience, Icahn School of Medicine at Mount Sinai, New York, New York, United States of America; Texas A&M University, UNITED STATES

## Abstract

Insight into motivational processes may be gained by examining measures of willingness to exert effort for rewards, which have been linked to neuropsychiatric symptoms of anhedonia and apathy. However, while much work has focused on the development of models of motivation based on classic tasks of externally-generated levels of effort for reward, there has been less focus on the question of self-generated motivation or volition. We developed a task that aims to capture separate measures of self-generated and externally-generated motivation, with two task variants for physical and cognitive effort, and sought to test and validate this measure in two populations of healthy volunteers (N = 27 and N = 28). Similar to previous reports, a sigmoid function represented a better overall fit to the effort-reward data than a linear or Weibull model. Individual sigmoid function shapes were governed by two free parameters: bias (the amount of reward needed for effort initiation) and reward insensitivity (the amount of increase in reward needed to accelerate effort expenditure). For both physical and cognitive effort, bias was higher in the self-generated condition, indicating reduced self-generated volitional effort initiation, compared to externally-generated effort initiation, across effort domains. Bias against initial effort initiation in the self-generated condition was related to a specific dimensional measure of anticipatory anhedonia. For physical effort only, reward insensitivity was also higher in the self-generated condition compared to the externally-generated motivation condition, indicating lower self-generated effort acceleration. This work provides a novel objective measure of self-generated motivation that may provide insights into mechanisms of anhedonia and related symptoms.

## Introduction

Neuropsychiatric symptoms of anhedonia and apathy can be conceptualized as diminished motivation for physical, cognitive or emotional activity. Insight into motivational processes may be gained by examining measures of willingness to exert effort for rewards, typically measured across species using forced-choice fixed or progressive ratio, accept/reject and dual-alternative tasks [1–3], for physical or cognitive effort [4]. Measures of motivation using these paradigms have been linked to depression [3] and have been incorporated as a critical domain within the Research Domain Criteria (RDoC) framework [5], acting as a trans-diagnostic construct that spans multiple symptom measurement domains.

However, while much work has focused on the development of models of motivation based on these classic tasks of externally-generated levels of effort for reward, there has been less focus on the question of self-generated motivation. Reinforcement learning models do not separate external and internal reward signals [6] and there is little convincing evidence yet to support dissociable neural mechanisms with neuroimaging [7]. Evidence for a neural distinction between self-generated and externally-generated motivation comes from patient studies with basal ganglia lesions who develop ‘psychic akinesia’, a difficulty with self-generated action initiation, but who show no difficulty in performing complex cognitive and physical tasks when prompted externally [8]. This specific difficulty in ‘volition’, or self-generated will to initiate action, supports a distinction from externally-generated motivation, an environmentally–stimulated prompt for action initiation. This volitional disruption has been associated with depressive symptoms [9]. Indeed, the overlapping symptom domains of apathy and anhedonia can be broken down into constituent cognitive processes, including an early option generation phase, which can be self-generated or environmentally-cued [1]. However, relatively few studies focus on this distinction [10] and there are no objective cognitive measures that dissociate self-generated volition from externally-generated motivation.

Here, we developed an objective task that aims to capture separate measures of self-generated and externally-generated motivation, based on a wealth of literature measuring motivation as willingness to exert effort for monetary rewards [3,11–13]. We sought to test and validate two variants of this measure (using physical or cognitive effort) in two populations of healthy volunteers. Similar to previous studies [13,14], we modeled reward by effort discount curves using a sigmoid function, which produces two major curve characteristics, reward insensitivity and bias, which can be directly compared between conditions. We primarily expected that self-generated and externally-generated motivation could be separately measured and dissociated within subjects. Secondarily, the dimensional construct of anhedonia was measured. Anhedonia can be broadly deconstructed into anticipatory and consummatory phases, for example by utilizing the Temporal Experience of Pleasure Scale (TEPS), which reliably produces a two-factor solution for anticipatory and consummatory aspects of anhedonia [15,16]. Anticipatory anhedonia has been linked to motivation in depressed individuals [17]. We therefore expected on an exploratory level that self-generated motivation, rather than externally-generated motivation would be associated with dimensional measures of anticipatory anhedonia in the healthy volunteer population.

## Methods

### Experiment 1

#### Participants

A community sample of healthy volunteers were recruited through the Depression and Anxiety Center at the Icahn School of Medicine at Mount Sinai, USA. Subjects were between the ages of 18–65 and were free from any current or lifetime psychiatric disorder as determined by the Structured Clinical Interview for DSM-V Axis Disorders (SCID-V) conducted by a trained rater [18]. Subjects were excluded if they had an unstable medical illness, history of neurological disease, neurodevelopmental/neurocognitive disorder, or positive urine toxicology test. All subjects completed cognitive testing. A subset of subjects completed a self-reported scale of anhedonia (TEPS) on the same day. All data was collected under Institutional Review Board (IRB)–approved written informed consent. All subjects were compensated for their time.

#### Internal-external motivation task (physical effort)

**Fig 1:**
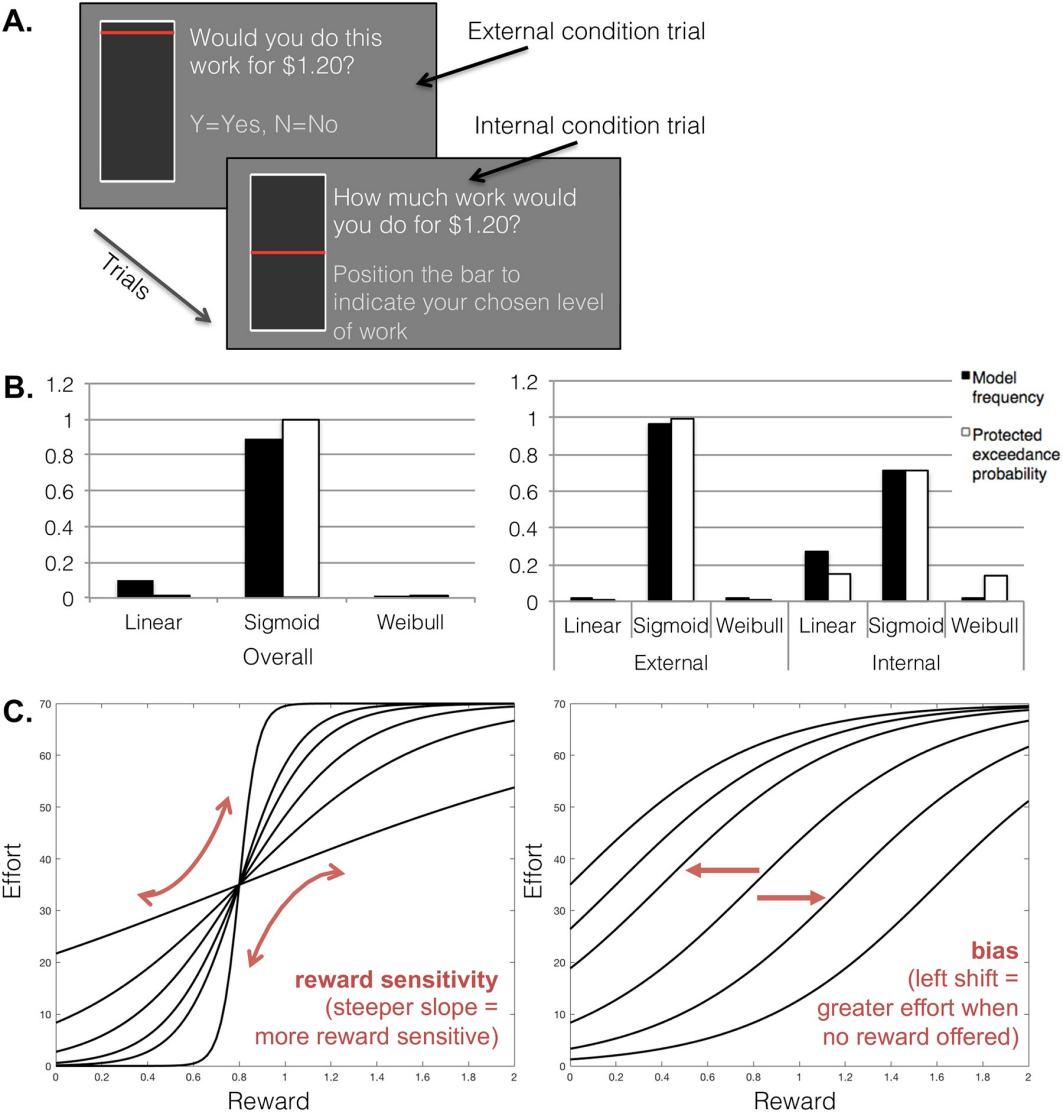
Differences between self-generated and externally-generated motivation during the internal-external motivation task. A: The internal-external motivation task (IMT) is a 15-minute effort-discounting decision-making task. The work involves keyboard button presses (3–70), which moves a visual bar to a target position for monetary reward ($0.25 to $2.00). In the external condition, subjects accept/reject with Y/N keyboard presses. In the internal condition, subjects position the bar to indicate the amount of work they would do. Thirty percent of trials lead to the work. B: A sigmoid function represented a better overall fit to the effort-reward data than a linear or Weibull model both when conditions were combined (left) and separated (right). C: Schematic depiction of the two free parameters computed after fitting the sigmoid model: reward sensitivity governing the slope of the function, which represents insensitivity of effortful responding to unit increases in offered reward, and bias (left-right translation parameter), which governs individual bias towards or away from expending effort.

The Internal-external Motivation Task (IMT) for physical effort is a 15-minute effort-discounting decision-making task programmed with PsychoPy software [19]. Subjects underwent training on the task in a self-paced manner before starting. During the task, subjects made decisions about how much work they would be willing to do for various amount of money. The work is a simple task of clicking the left and right keyboard buttons quickly to move a visual bar up the computer screen to a target position, which requires between 3 and 70 button presses (Fig 1A). The money ranges from $0.25 to $2.00. In the external condition, subjects respond by pressing Y (yes) or N (no) on the keyboard. In the internal condition, subjects position the bar to indicate the maximum amount of work they would do for each reward (Fig 1A). This was repeated for 128 trials and provides a measure of how willing an individual is to perform physical effort for rewards. Internal and external condition trials were interleaved and response times were self-paced. Thirty percent of trials lead to the work. No feedback was provided throughout the task. Subjects were instructed that the more they ‘work’ the more likely they are to get the money, that there was a hidden threshold of amount of work needed throughout the task, and at the end of the task, one of their responses will be randomly chosen and paid out. In actuality, all subjects received the maximum amount at the end of the study. The internal condition captures self-generated motivation or volition, whereas the external condition captures externally-generated motivation.

### Experiment 2

A college student sample of healthy volunteers was recruited through the Brighton and Sussex Medical School, UK. Subjects were above the age of 18 and free of any medical disorders or current or lifetime psychiatric disorder as determined by the Mini-Mental State Examination [20]. All subjects performed the cognitive task and the Beck Depression Inventory (BDI) [21]. The study was approved by the University of Cambridge research ethics committee and all subjects were compensated for their time.

#### Internal-external motivation task (cognitive effort)

For the cognitive effort variant of the IMT, participants were first asked to perform a version of a mental effort task (Serial Three’s) [22,23], in which they were asked to count backwards from 200 in 3’s aloud for 1 minute. This experiential effortful counting task was repeated 3 times. Subjects were then asked to indicate the amount of time in minutes they would be willing to spend performing this effort task for increasing amounts of monetary reward (£1-£500) via questionnaire. Similar to the IMT, in the external condition participants were provided with specified amounts of time (1–200 minutes) to which they responded with Y (yes) or N (no). The maximum number of effort for each reward was selected and entered into subsequent analyses. In the internal condition, participants were free to enter any amount of minutes for the same monetary magnitudes. However, in the internal condition, there was no upper boundary of minutes and several participants indicated more than 200 minutes for reward magnitudes higher than £200. Therefore, these magnitudes were excluded from the analysis in order to allow direct comparisons between the internal and external conditions.

### Modeling and statistics

In order to quantify the relationship between effort and reward in our samples, and determine how this might be affected by internally vs externally generated motivation conditions, we fit several candidate psychometric functions to task data. Linear, sigmoid and Weibull functions were fit to the effort-by-reward discount curves for each condition for subject using the variational Bayes approach to model inversion implemented in the VBA toolbox [24] (available at mbb-team.github.io/VBA-toolbox), run in MATLAB R2019a. In order to reduce the likelihood of outlier values for any individual parameter estimates, curve fitting was performed under a mixed-effects framework. Specifically, after the first round of model inversion, the individual posterior parameter estimates were used to approximate the population distribution these values were drawn from (assumed to be Gaussian), which was then used as prior for the next round of inference, a process that was repeated until convergence (no further gain in group-level likelihood). As data from both externally and internally generated motivation conditions was included during this process, this procedure is likely to be conservative with respect to differences in parameter estimates between experimental conditions (as all individual parameter estimates will be shifted towards the group mean).

The functions used to relate effort expended (y) against reward offered (x) were:

1. Linear:
  y=mx+c
  where m is the gradient, and c is the y intercept parameter
2. Sigmoid:
  y=c*1./(1+exp(−x−bias)/sigma)))
  where the bias parameter governs the left-right translation of the function (bias against exerting effort, for a given amount of reward), sigma is the gradient governing reward sensitivity (increase in effort per increase in unit reward offered).
3. Weibull:
  y=A*(1−2.^(−x.*L).^S))
  where L is the latency of the function (governing minimum reward necessary to begin expending effort), S governs the abruptness of the function (reward sensitivity), and A controls the asymptote (ceiling on amount of effort that will be extended, for any reward offered).

The best fitting function relating effort to reward was then selected using random-effects Bayesian model comparison [25]. The critical output metric for this approach to model comparison is exceedance probability, or the likelihood that that particular model is more frequent than all other models in the comparison set. Protected exceedance probability is an extension of this metric, corrected for the possibility that observed differences in model evidences are due to chance [25]. That the same function provided the best fit to data across internally and externally generated motivation trials was then confirmed by performing between-conditions random-effects Bayesian model selection on the same data.

In order to test the reliability of model-based parameter estimates, simulation-recovery analysis was performed. For each experiment, *N* = 1000 random samples were drawn from the group-level posterior distribution of each parameter. Task data were then simulated for each set of randomly sampled parameters using the VBA toolbox function VBA_simulate (using the same number of observations and reward levels as in the experimental data for each experiment). Model inversion was then applied to the simulated data for each sample. Finally, actual and recovered (estimated) parameter values were compared using bivariate Pearson correlations.

For both studies, parameter estimates from the winning model were derived for each condition for each subject and entered into paired-samples *t*-tests to compare between conditions. These parameters were additionally correlated (Pearson) with self-reported measures of pleasure anticipation and consumption (TEPS), inverse dimensional markers of anticipatory and consummatory anhedonia, respectively [15,16].

## Results

### Experiment 1

Twenty-seven healthy volunteers recruited from the community completed the IMT for physical effort. Data from 1 subject was removed due to technical issues, leaving 26 subjects (age = 40.18 ± 10.1; 10 female).

Similar to previous reports [13,14], a sigmoid function represented a better overall fit to the effort-reward data than a linear or Weibull model (protected exceedance probability > 0.999, estimated model frequency = 0.894, Fig 1B). Between-conditions Bayesian model selection confirmed that the best fitting model did not differ across externally and internally generated conditions (protected exceedance probability for between-conditions stability = 1.00). The sigmoid model provided a good account of individual effort-reward functions across participants (external condition mean *r*^2^ = 0.767 ± 0.20, internal condition mean *r*^2^ = 0.805 ± 0.22). See S1 Table for individual model fits.

Individual sigmoid function shapes were governed by two free parameters: an inverse gradient parameter governing the slope of the function, which represents insensitivity of effortful responding to unit increases in offered reward, and a bias (left-right translation parameter), which governs individual bias towards or away from expending effort at a given reward level (Fig 1C). See S1 and S2 Figs for further information on the best-fitting parameters.

Reliability of the individual parameter estimates derived from the sigmoid model was assessed for each experiment using simulation-recovery analysis (see Methods). For Experiment 1, the correlation between simulated and recovered parameter estimates for *N* = 1000 randomly drawn samples was *r* = 0.899 for the reward sensitivity (gradient) parameter, and *r* = 0.530 for the bias parameter. For Experiment 2, the correlations were *r* = 0.882 and *r* = 0.741 for the reward sensitivity and bias parameters, respectively. Therefore, parameter estimation procedures demonstrated acceptable to good reliability across datasets.

**Fig 2:**
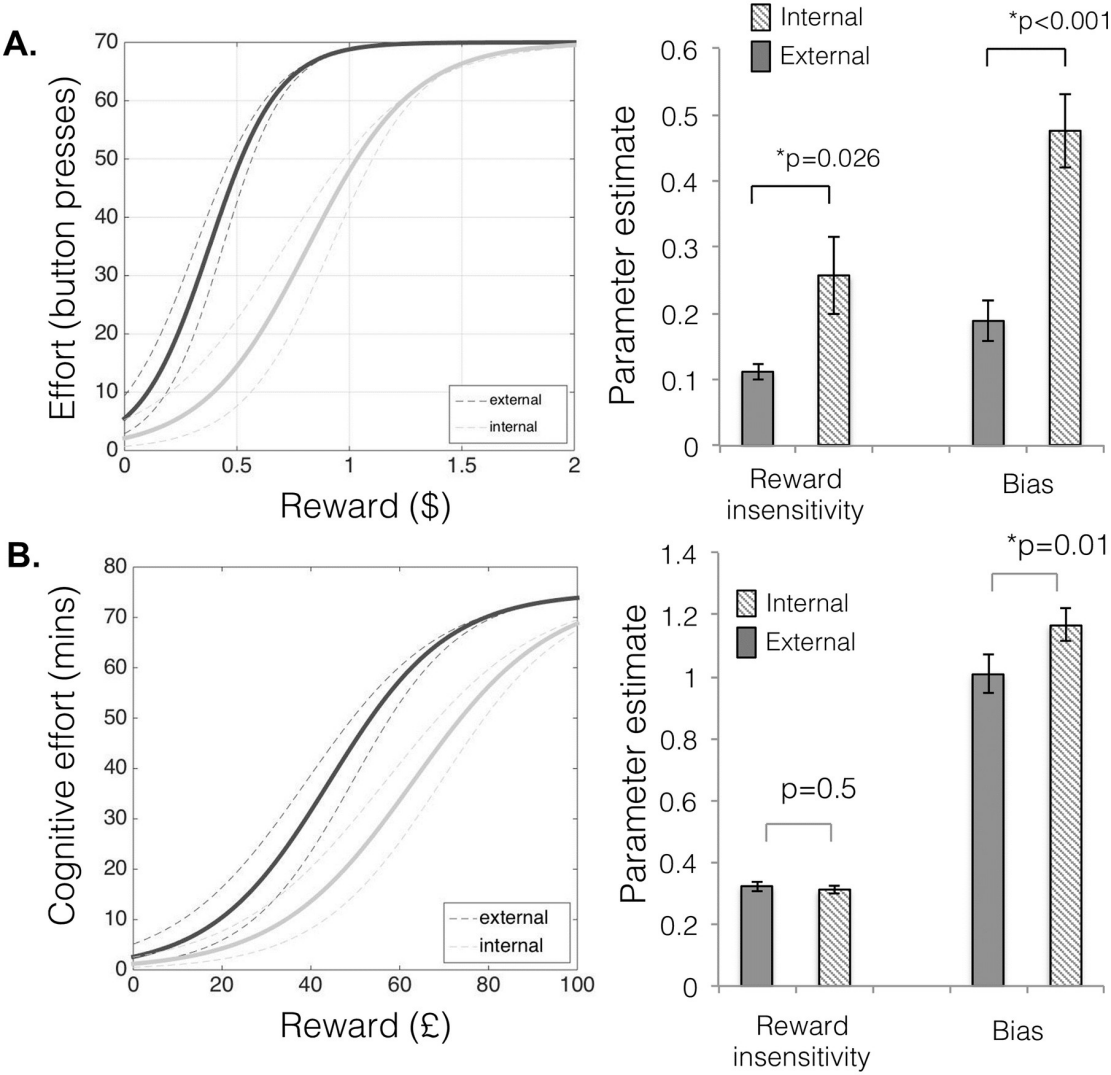
Differences between self-generated and externally-generated motivation during the internal-external motivation task for physical and cognitive effort. **A**. Using the IMT with physical effort, both model parameters (reward insensitivity and bias) were significantly different between conditions (reward insensitivity, t_(25)_ = 2.36, p = 0.026; bias, t_(25)_ = 5.77, p = 5x10^-6^). **B**. For cognitive effort, there were significant differences between conditions in bias away from expending effort (t_(27)_ = -2.703, p = 0.012), while there were no differences in reward insensitivity (t_(27)_ = 0.654, p = 0.519).

There were significant differences between conditions in both reward insensitivity (inverse gradient, paired samples t-test, t_(25)_ = 2.36, p = 0.026) and bias away from expending effort (t_(25)_ = 5.77, p = 5x10^-6^) parameters, in which the internal condition was associated with higher reward insensitivity and greater bias away from effort expenditure compared to the external condition (Fig 2A). Age was not correlated with any of the parameters and there were no differences related to sex (p’s>0.05).

**Fig 3:**
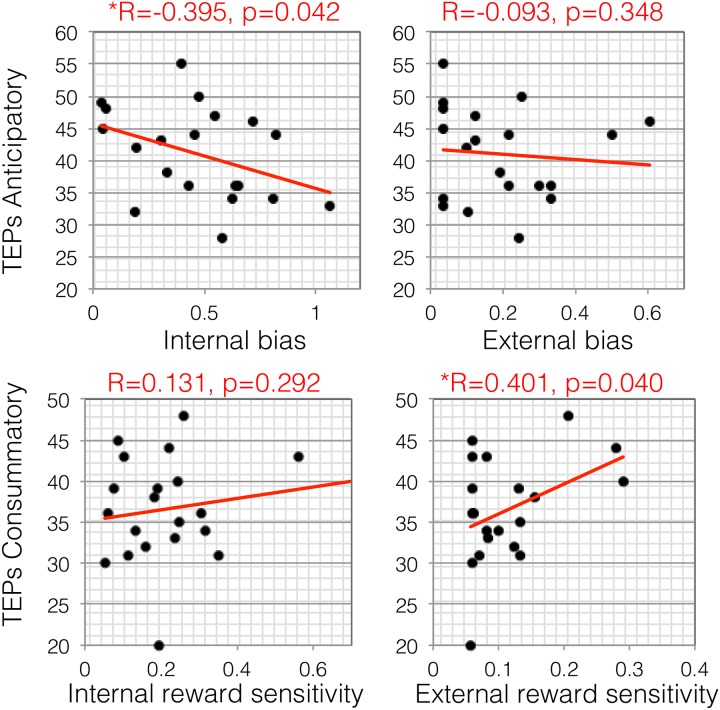
Objective measures of self-generated and externally-generated motivation plotted against self-reported anticipatory and consummatory anhedonia. Self-generated motivation for physical effort, as measured by bias away from effort expenditure in the internal condition, was correlated with pleasure anticipation (R = -0.395, p = 0.042) in N = 20 subjects. Reward insensitivity was associated with pleasure consummation in the external condition (R = 0.401, p = 0.040).

Self-generated motivation, as measured by bias away from effort expenditure in the internal condition, was correlated with pleasure anticipation (R = -0.395, p = 0.042), whereas externally-generated motivation was not (R = -0.93, p = 0.348; difference in correlations: Z = 4.99, p = 5.9x10^-7^) in N = 20 subjects who completed the self-reported measure of pleasure experience (Fig 3). Interestingly, the reverse was apparent for reward insensitivity, which was associated with pleasure consummation in the external condition (R = 0.401, p = 0.040), but not in the internal condition (R = -0.046, p = 0.423; difference in correlations: Z = 1.54, p = 0.12). All measures across conditions were not correlated with recent caffeine intake, approximate annual income or how difficult subjects reported the button pressing (p’s>0.05).

### Experiment 2

A college-based sample of 28 healthy volunteers also completed the IMT for cognitive effort (age = 20.71 ±1.8; 22 female; BDI = 9.8±10.2). For comparison with data from Experiment 1, sigmoid curves were fit to individual choice-cognitive effort functions (external condition mean *r*^2^ = 0.741 ± 0.25, internal condition mean *r*^2^ = 0.738 ± 0.27).

Similar to *Experiment 1*, there were significant differences between conditions in bias away from expending effort (t_(27)_ = -2.703, p = 0.012, Fig 2B), whereby the internal condition was associated with greater bias away from effort expenditure compared to the external condition. However, there was no difference in reward sensitivity (t_(27)_ = 0.654, p = 0.519, Fig 2B) suggesting robust differences between self-generated and externally-generated *initiation* rather than *acceleration* of effort, across effort domains. There was no relationship between these variables and BDI (p’s>0.05).

## Discussion

This work aimed to present a novel objective task that is able to dissociate self-generated and externally-generated motivation in humans. We show that healthy individuals were willing to exert more effort when options were explicit or externally-generated compared to when the degree of effort was self-generated. Both the amount of reward needed for effort initiation (bias) and the amount of increase in reward needed to accelerate effort expenditure (reward insensitivity) were higher in the self-generated condition for physical effort. Bias against initial effort initiation was also observed for cognitive effort, thereby indicating a persistent bias across two different samples of healthy volunteers, for both small and large monetary rewards and across both cognitive and physical effort domains. In a secondary exploratory analysis, this bias against physical effort initiation was related to a specific measure of anticipatory anhedonia, rather than consummatory anhedonia. This work provides a novel objective measure of self-generated motivation that may provide insights into mechanisms of anhedonia and apathy.

The current study assessed both cognitive and physical effort. Although distinctions can be made between neural correlates of motor (primary sensorimotor cortex) and cognitive (inferior parietal and dorsolateral prefrontal cortices) effort, one study has demonstrated that performance on both can be predicted by an equal motivational system represented by the ventral striatum [26]. In the same study, no motivational system could be distinguished that singularly serves either cognitive or motoric effort, but rather commonly drives both [26]. Indeed, ventral striatal dopamine prediction error [27,28] has been suggested to act not as a reward-updating signal but rather as a motivational signal [29], with the mesolimbic dopaminergic system being linked to ‘wanting’ rather than ‘liking’ [7,30] and to effort-based decision-making [31]. Interestingly, a recent study [32] provides an update on the traditional reinforcement-learning properties of midbrain dopamine neuron activity by demonstrating encoding of distributions of outcome probabilities, rather than a single mean prediction error value per se. However, how this summates or feeds forward towards action choice and initiation is yet to be determined. On a broader scale, via functional interactions, the ventral striatum can recruit the putamen for motor effort and caudate for cognitive effort [26]. Indeed, modulation of ventral striatal function in non-human primates reduces the amount of self-generated actions to retrieve rewards [33] and dopaminergic function determines both value and vigor associated with chosen action [34,35]. We would therefore expect that while both forms of self-generated and externally generated motivation would recruit ventral striatal dopamine function, it might be preferentially recruited during self-generated motivational choices. Interestingly, stimulation of dopaminergic projections from the ventral tegmental area (VTA) to the ventral striatum and medial prefrontal cortex seem to increase self-generated motivation or energy [36], suggesting a neural pathway by which self-driven motivation could be generated.

While bias against initial effort initiation was higher in the self-generated condition compared to the externally-generated condition across both effort domains, reward insensitivity was only higher for self-generated choices in the physical effort domain. Additionally, externally-generated physical effort acceleration was related to reward consummation. This may be partly explained by the differing neural computational mechanisms supporting each type of effort. Dopamine modulates physical effort allocation but has less of a role in cognitive effort allocation [37]. Thus, self-generated effort acceleration in the physical effort domain may be regulated by dopaminergic and overall intrinsic motivational tone related to outcome, whereas cognitive effort acceleration may be less governed by underlying dopaminergic tone. Indeed, the computation of cognitive demand is performed by a separate fronto-parietal network, rather than a more distributed motor-striatal network, which underlies computation of physical demand [26]. The lack of difference between self-generated and externally-generated effort acceleration for cognitive effort may therefore reflect a lower reliance on intrinsic motivational tone or internal scaling functions for cognitive effort allocation governed by dopaminergic systems. Future studies should directly test this using functional MRI to measuring striatal and whole brain activation during cognitive and physical effort allocation for increasing rewards.

Overall, the current study provides a means of distinguishing between self-generated and externally generated motivation for physical and cognitive effort. Both of these constructs of motivation are considered ‘extrinsic’ rather than ‘intrinsic’ as they utilize an external reward, rather than being inherently interesting or enjoyable [38]. Intrinsic motivation is often conceptualized as motivation for learning, contentment or achievement, driven by the perceived value of the work at hand, rather than motivation in terms of effort for an explicit external reward. In the current study, individuals were consistently less willing to work in the self-generated condition, which may reflect the behavioral undermining effect [39] whereby rewards dampen intrinsic motivation for task engagement. Choice of higher amounts of effort in the externally generated condition may alternatively be explained by the status quo bias [40], wherein individuals adhere to an explicitly generated option.

This work may have implications for characterizing, diagnosing and tracking treatment outcomes of neuropsychiatric disorders characterized by avolition, apathy and anhedonia. These terms are often considered as unitary constructs and are listed as single symptoms. However, they cover a range of potential processes, from aberrant self-driven option generation, to deficits in action initiation, to disruption in reinforcement learning [1]. Considering the construct of anhedonia, which relates to lack of pleasurable experience, recent studies suggest that pleasurable experience per se may be intact in patients with depression and schizophrenia [1, 41], suggesting that more volitional or motivational aspects of behavioral drive may be more relevant. By deconstructing these broad symptom domains into constituent components, more fine-grained tools for diagnostics, treatment tracking and intervention can be developed for individualized precision medicine.

The current study has several limitations. While all participants were recruited in the same manner, the motivational influence of monetary rewards may vary depending on individual differences in family income or salary. However, we did not observe any relationship between self-generated or externally-generated motivation and annual salary. A proportion of subjects in the external condition consistently accepted the maximum level of effort for the higher reward levels, suggesting a potential ceiling effect. However, that this was observed across both experiments, where reward levels differed in magnitude, suggests that this might be a consistent trait and not related to reward magnitude or availability. This certainly warrants further exploration in follow-up studies. Another limitation was that the measures of both physical and cognitive effort were not collected in the same cohort of subjects and the same specific self-report scales of anhedonia were not collected in both cohorts. The ages of the subjects, reward amounts and currency differed between the two studies, which while being a limitation, does underscore the consistency of the finding of reduced self-generated bias across ages, reward scales and currency systems. Further studies that comprehensively examine these constructs in a single larger sample of healthy controls are warranted.

In conclusion, this work demonstrates an objective task that separates measures of self-generated and externally-generated motivation, illustrating greater bias away from initial effort expenditure when effort is self-generated, across both cognitive and physical effort. This bias against self-generated physical effort initiation was related to anticipatory anhedonia, highlighting an objective measure of self-generated motivation or volition that may provide novel insights into mechanisms of anhedonia.

## Supporting information

S1 Fig A. Sigmoid model fit curves for each subject for external and internal task conditions for Experiment 1. B. Individual model fit plots for each participant. Red circles represent the raw (input) data for that participant. The blue line is the curve generated using the mean posterior parameter values for that participant (sigmoid model).
(DOCX)

S2 Fig A. Sigmoid model fit curves for each subject for external and internal task conditions for Experiment 2. B. Individual model fit plots for each participant. Red circles represent the raw (input) data for that participant. The blue line is the curve generated using the mean posterior parameter values for that participant (sigmoid model).
(DOCX)

S1 Table Means and ranges of best-fitting parameters for the sigmoid model fit for each condition for each experiment. SD = standard deviation.
(DOCX)
