# Supplementary material for: Dissociating self-generated volition from externally-generated motivation
Source: PLoS One. 2020 May 19;15(5):e0232949. doi: 10.1371/journal.pone.0232949 (PMC7236980; doi:10.1371/journal.pone.0232949)
Supplement: S2 Fig — A. Sigmoid model fit curves for each subject for external and internal task conditions for Experiment 2. B. Individual model fit plots for each participant. Red circles represent the raw (input) data for that participant. The blue line is the curve generated using the mean posterior parameter values for that participant (sigmoid model). (DOCX) [file pone.0232949.s002.docx]

**Supplemental Figure 2.**

**Supplemental Figure 2.** A. Sigmoid model fit curves for each subject for external and internal task conditions for Experiment 2. B. Individual model fit plots for each participant. Red circles represent the raw (input) data for that participant. The blue line is the curve generated using the mean posterior parameter values for that participant (sigmoid model).
