# Supplementary material for: Dissociating self-generated volition from externally-generated motivation
Source: PLoS One. 2020 May 19;15(5):e0232949. doi: 10.1371/journal.pone.0232949 (PMC7236980; doi:10.1371/journal.pone.0232949)
Supplement: S1 Table — SD = standard deviation. (DOCX) [file pone.0232949.s003.docx]

**Supplemental Table 1.**

|  | **External** |  | **Internal** |  |
| --- | --- | --- | --- | --- |
|  | **Mean** | **Range** | **Mean** | **Range** |
| **Experiment 1** |  |  |  |  |
| Bias | 11.3 (SD 5.1) | 3.2‑19.7 | 6.25 (SD 4.8) | 0.60­‑19.4 |
| Reward Sensitivity | 0.492 (SD 0.26) | 0.01-1.1 | 0.191 (SD 0.17) | 0.01-0.6 |
| **Experiment 2** |  |  |  |  |
| Bias | 7.52 (SD 4.3) | 2.8‑20.1 | 6.52 (SD 3.3) | 2.7‑12.7 |
| Reward Sensitivity | 0.442 (SD 0.28) | 0.05-1.1 | 0.631 (SD 0.33) | 0.10-1.1 |

**Supplemental Table 1.** Means and ranges of best-fitting parameters for the sigmoid model fit for each condition for each experiment. SD= standard deviation.
